# Alcohol-related liver disease (ALD): current perspectives on pathogenesis, therapeutic strategies, and animal models

**DOI:** 10.3389/fphar.2024.1432480

**Published:** 2024-11-28

**Authors:** Xiao Hong, Shuo Huang, He Jiang, Qing Ma, Jiang Qiu, Qihan Luo, Chunlu Cao, Yiyang Xu, Fuzhe Chen, Yufan Chen, Chunfeng Sun, Haozhe Fu, Yiming Liu, Changyu Li, Fangming Chen, Ping Qiu

**Affiliations:** ^1^ School of Pharmaceutical Sciences, Zhejiang Chinese Medical University, Hangzhou, China; ^2^ School of Basic Medical Science, Zhejiang Chinese Medical University, Hangzhou, China; ^3^ The Second School of Clinical Medicine, Zhejiang Chinese Medical University, Hangzhou, China; ^4^ The First School of Clinical Medicine, Zhejiang Chinese Medical University, Hangzhou, China; ^5^ Department of Medicine, Hangzhou Normal University, Hangzhou, China; ^6^ The First People’s Hospital of Xiaoshan District, Xiaoshan Affiliated Hospital of Wenzhou Medical University, Hangzhou, China; ^7^ Academy of Chinese Medical Science, Zhejiang Chinese Medical University, Hangzhou, China

**Keywords:** alcohol-related liver disease, cell death patterns, intestinal flora, immune response, molecular mechanisms, therapeutic strategy, animal models

## Abstract

Alcohol-related liver disease (ALD) is a major cause of morbidity and mortality worldwide. It encompasses conditions such as fatty liver, alcoholic hepatitis, chronic hepatitis with liver fibrosis or cirrhosis, and hepatocellular carcinoma. Numerous recent studies have demonstrated the critical role of oxidative stress, abnormal lipid metabolism, endoplasmic reticulum stress, various forms of cell death (including apoptosis, necroptosis, and ferroptosis), intestinal microbiota dysbiosis, liver immune response, cell autophagy, and epigenetic abnormalities in the pathogenesis of ALD. Currently, abstinence, corticosteroids, and nutritional therapy are the traditional therapeutic interventions for ALD. Emerging therapies for ALD mainly include the blockade of inflammatory pathways, the promotion of liver regeneration, and the restoration of normal microbiota. Summarizing the advances in animal models of ALD will facilitate a more systematic investigation of the pathogenesis of ALD and the exploration of therapeutic targets. This review summarizes the latest insight into the pathogenesis and molecular mechanisms of ALD, as well as the pros and cons of ALD rodent models, providing a basis for further research on therapeutic strategies for ALD.

## 1 Introduction

Alcohol-related liver disease (ALD) represents a major public health problem worldwide. Alcohol consumption is the leading cause of chronic liver disease, potentially leading to conditions such as fatty liver, alcohol-related hepatitis (AH), liver cirrhosis, and/or liver cancer (Huang et al., 2023). A recent study calculated the proportion and number of patients with liver disease both nationally and internationally up to 2020. The data indicated that the number of patients with ALD in China was second to the number of hepatitis B cases, reaching 62 million (Xiao et al., 2019). There are approximately 2.3 billion alcohol consumers globally, with more than half of them residing in Western countries, including the United States. The incidence of diseases and fatalities stemming from alcohol consumption is also increasing each year. According to the World Health Organization, alcohol-related deaths have surged to three million (Avila et al., 2020), accounting for 5% of the global mortality in 2016. Deaths attributable to alcohol-related digestive system diseases account for 21% of all disease-related deaths. In the United States, the incidence of alcohol-induced liver cirrhosis increased by 43% over 7 years, and the number of deaths resulting from alcohol increased by 2.1 times between 2000 and 2019 (Niu et al., 2023; Bajaj, 2019). AH is the most severe acute form of ALD, characterized by severe jaundice and liver failure. Severe AH is associated with very high short-term mortality, with a 30-day mortality rate of 20%–50% (Saberi et al., 2016).

Although ALD has a profound impact on public health, it is an under-researched area. Currently, the pathogenesis of ALD remains unclear. Abstinence, corticosteroids, and nutritional supplementation have been considered conventional clinical therapeutic strategies for ALD. Therefore, current researchers are focusing on exploring promising therapeutic strategies, such as inhibiting pro-inflammatory processes, promoting liver regeneration, and modulating gut microbiota homeostasis. This paper summarizes recent studies on the molecular mechanisms underlying the pathogenesis of ALD. This review also provides an overview of current and emerging therapeutic strategies for treating ALD and recent advances in rodent models of binge ethanol administration, which aids in replicating various characteristics of ALD progression.

## 2 Molecular mechanisms of ALD

The pathogenesis of ALD is intricate and encompasses diverse pathological processes, including oxidative stress, abnormal lipid metabolism, endoplasmic reticulum stress, autophagy, various modes of cell death (including pyroptosis, necroptosis, and ferroptosis), gut microbiota dysbiosis, hepatic immune response and epigenetic abnormalities.

### 2.1 Oxidative stress and ethanol metabolites

Alcohol is metabolized in the liver by alcohol dehydrogenase (ADH), cytochrome P450 2E1 (CYP2E1), and catalase, concurrently generating reactive oxygen species (ROS) and leading to oxidative stress (Yang et al., 2022). The induction of CYP2E1 can stimulate the transport of reduced NADH to the mitochondria, which increases electron leakage from the hepatocyte mitochondrial respiratory chain and ROS generation. Toxic metabolites derived from CYP2E1 recruit leukocytes and hepatic stellate cells (HSCs), exacerbating the hepatic inflammatory response and fibrotic processes (Michalak et al., 2021). CYP2E1-deficient mice exhibited attenuated hepatic oxidative stress and steatosis after chronic alcohol feeding. Hepatic steatosis is associated with mitochondrial dysfunction and excessive mitochondrial ROS production. Recently, a large number of genes have been involved in the progression of ALD by regulating liver redox homeostasis. Zhang et al. (2021) suggested that the absence of the Fas-activated serine/threonine kinase (FASTK) gene may inhibit the ethanol-induced phosphorylation of human antigen R (HuR) and dissociation of the HuR-sirtuin 1 mRNA complex. This increases the stability of the complex, lowering ROS production and maintaining the stability of the liver antioxidant system after alcohol intake. Hong et al. (2022) found that knocking out Dnajc10 (ERdj5) exacerbated ethanol-triggered liver damage in mice. This could be attributed to the inhibition of the antioxidant regulatory nuclear factor E2-related factor 2 (Nrf2) and the subsequent decrease in glutathione content, which exacerbated oxidative stress and hepatic steatosis. Therefore, inhibiting FASTK or promoting Dnajc10 gene expression can be effective strategies to alleviate ethanol-induced oxidative stress and protect liver cells.

Common liver tissue damage is mainly due to the accumulation of toxic aldehydes during the lipid peroxidation process under oxidative stress. The production of acetaldehyde promotes glutathione depletion, free radical-mediated toxicity, and immune reactions generated by pro-inflammatory cytokines (Yang et al., 2022). Due to the instability of aldehyde molecules, they rapidly react with cellular components to form adducts, ultimately leading to cytotoxicity. Furthermore, ROS can interact with various lipids to generate lipid peroxidation products such as malondialdehyde and 4-hydroxynonenal. These substances can combine with proteins to form acetaldehyde adducts, triggering immune responses and exacerbating inflammation and fibrosis in ALD patients (Michalak et al., 2021). Accumulated toxic aldehydes in the liver can be effectively metabolized by mitochondrial aldehyde dehydrogenase 2 (ALDH2), thereby alleviating various liver diseases. Mutations in the ALDH2 gene lead to impaired ALDH2 enzymatic activity, exacerbating the progression of liver diseases. Wang et al. found that 45% of East Asians have a deficiency in ALDH2 enzymatic activity, leading to ALD and increasing the risk of hepatocellular carcinoma (HCC) (Wang et al., 2021). Gao et al. reported that the expression levels of ALDH2 decreased in mice after long-term alcohol intake, leading to the accumulation of toxic aldehydes and accelerating liver inflammation and fibrosis (Gao et al., 2019b). Simultaneously, another study confirmed that ALDH2-deficient mice treated with ethanol were more susceptible to ALDs, including AH, liver fibrosis, and HCC (Wu et al., 2023b). High levels of acetaldehyde can alter intestinal permeability and microbiota homeostasis, leading to direct liver cell damage (Patel et al., 2022a). Due to the toxic effects of acetaldehyde, substantial evidence shows that the ALDH2*2 polymorphism is associated with a higher risk of liver cancer (Seo et al., 2019). Alcohol increases the production of reactive nitrogen species and nitrosative stress, disrupting the balance of the oxidative and antioxidant systems, thus exacerbating the progression of liver diseases (Loguercio and Federico, 2003).

### 2.2 Lipid deposition in hepatocytes

Drinking alcohol can alter lipid metabolism through multiple mechanisms. Chronic alcohol ingestion increases the ratio of attenuated NAD/oxidized NAD (NADH/NAD+) in hepatocytes, disrupting the mitochondrial beta-oxidation of fatty acids and leading to liver steatosis (Masia et al., 2018). In addition, excessive alcohol intake enhances hepatic protein levels of SREBP1c, a transcription factor that elevates the expression of genes involved in lipid synthesis (Hu et al., 2022). Chronic ethanol intake also suppresses the activity of peroxisome proliferator-activated receptor-alpha (PPARα), which is a nuclear hormone receptor that upregulates the expression of many genes involved in the transport and beta-oxidation of free fatty acids (FFAs), including the gene encoding carnitine palmitoyltransferase 1 (CPT1) in mitochondria (Zhang et al., 2018a). Available evidence has revealed that lipin-1 plays a critical role as an Mg2+-dependent phosphatidic acid phosphatase in lipid homeostasis, promoting the accumulation of triglycerides in hepatocytes and alcohol-related steatohepatitis (Hu et al., 2013; Zhang et al., 2018b). Apart from altering lipid metabolism, alcohol intake also affects the mobilization and clearance of fatty acids. Increasingly, studies have suggested that long-term alcohol ingestion triggers the lipolysis of adipose tissue, leading to an increase in circulating FFAs and subsequent hepatocellular lipid accumulation (Mathur et al., 2023). Recent research determined that chronic alcohol intake inhibited the activation of mammalian target of rapamycin complex 1 (mTORC1), leading to an uncoupling status between lipolysis and the browning/thermogenesis of white adipose tissue (WAT), and thus exacerbating hepatic lipid accumulation, inflammation, and fibrosis in the development and progression of ALD (Song et al., 2023).

### 2.3 Endoplasmic reticulum stress (ERS)

**FIGURE 1:**
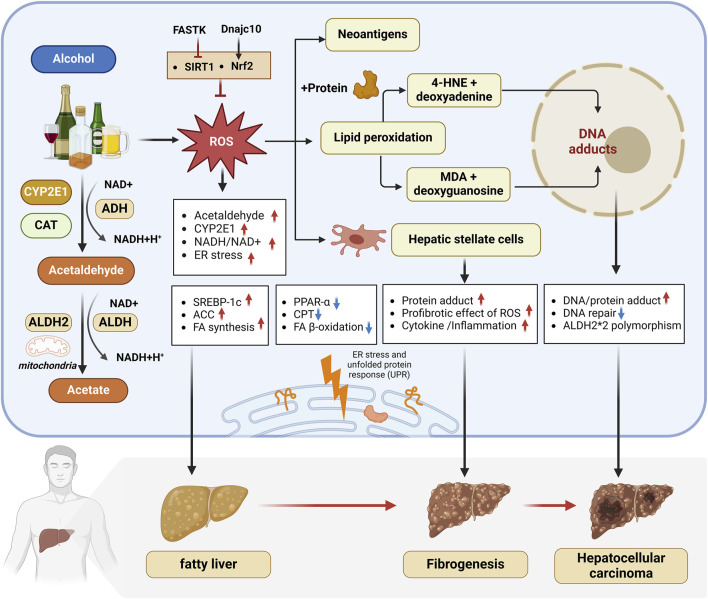
Oxidative stress and ERS have been recognized as crucial mechanisms in ALD pathogenesis. 1) Alcohol metabolism in the liver is mainly carried out through ADH, CYP2E1 and CAT, which produces ROS and triggers oxidative stress. 2) Long-term drinking increases the NADH/NAD + ratio, promotes the expression of lipid synthesis-related genes SREBP1c and ACC, inhibits the expression of mitochondrial fatty acid β-oxidation genes PPARα and CPT, and leads to fatty liver. 3) Excessive ROS induces lipid peroxidation, and the toxic aldehydes such as 4-HNE and MDA produced can form adducts, mediate immune responses and cause liver cell damage, and synergize with ROS to aggravate inflammation and fibrosis in ALD patients and increase the risk of liver cancer. 4) FASTK inhibits SIRT1 expression to aggravate ALD, while Dnajc10 promotes Nrf2 to alleviate ALD. 5) ALDH2 can effectively metabolize toxic aldehydes and slow down the progression of liver disease. ALDH2*2 gene mutations are associated with a higher risk of liver cancer. 6) Alcohol intake can induce oxidative stress, leading to an imbalance in the unfolded protein response in the endoplasmic reticulum (ER), aggravating ER stress and promoting the occurrence and development of ALD. abbreviation: 4-HNE, 4-Hydroxy-2-nonenal; ACC, Acetyl-CoA Carboxylase Alpha; ADH, Alcohol Dehydrogenase; ALDH, Aldehyde dehydrogenase; ALDH2*2, a genetic variant of the ALDH2 gene; ALDH2, Aldehyde dehydrogenase 2; CPT, Carnitine Palmitoyltransferase 1A; CYP2E1, Cytochrome P450 2E1; DNAJC10, DnaJ Heat Shock Protein Family (Hsp40) Member C10; FA, Fatty acid; FASTK, Fas Activated Serine/Threonine Kinase; FFAs, Free fatty acids; HCC, Hepatocellular Carcinoma; MDA, Malondialdehyde; NADH/NAD+: Nicotinamide adenine dinucleotide, oxidized form/Nicotinamide adenine dinucleotide; NRF2, Nuclear factor erythroid 2–related factor 2; PPAR-α: Peroxisome Proliferator Activated Receptor Alpha; ROS, Reactive Oxygen Species; SIRT1, Sirtuin 1; SREBP-1C, Sterol Regulatory Element Binding Transcription Factor 1.

ERS is a cellular stress response initiated by cells as a self-protective mechanism. When unfolded or incorrectly folded proteins are present in the cell, ERS is activated to diminish the concentration of these abnormal proteins and prevent their aggregation. However, the excessive activation of ERS can also be detrimental to cells. Ethanol consumption induces oxidative stress, leading to an oxidative environment in the endoplasmic reticulum (ER), which results in an imbalance in the unfolded protein response, heightened ERS, and the induction of endogenous cellular apoptosis, ultimately damaging liver cells (Koo et al., 2016). The sirtuin family, a class of deacetylases in the body, is associated with ALD. Xin et al. demonstrated that the overexpression of sirtuin 6 (SIRT6) could mitigate ERS in ALD mice, thereby reducing alcohol-induced liver damage (Xin et al., 2021). This indicates that SIRT6 acts as an inhibitor of alcohol-induced ERS and can prevent ALD by inhibiting ERS. Wang et al. suggested that the gamma-aminobutyric acid signaling system in the liver may inhibit the immunoglobulin-regulated enhancer 1/apoptosis signal-regulating kinase 1/c-Jun N-terminal kinase (IRE1/ASK1/JNK) cell apoptosis pathway triggered by ERS (Wang et al., 2018). This inhibition potentially reduces liver cell death and protects the liver. In general, oxidative stress and ERS are crucial factors in the pathological progression of ALD and the main mechanisms are shown in Figure 1.

### 2.4 Autophagy

Cellular autophagy is the process through which eukaryotic cells degrade excess or damaged substances within the cell using organelles such as lysosomes. It plays a crucial role in maintaining the homeostatic balance of cells and organisms. However, this autophagy is also regulated by alcohol and is associated with both the acute and chronic intake of alcohol. *In vivo* and *in vitro* evidence confirms that acute alcohol intake can stimulate autophagy in hepatocytes, which may be directly related to the activation of the CYP2E1 pathway and the generation of ROS (Salete-Granado et al., 2023). Eid et al. demonstrated that acute alcohol exposure can trigger type 2 mitochondrial autophagy in liver cells through the tensin homolog-induced putative kinase 1/Parkin/microtubule-associated protein light chain 3 (PINK1/Parkin/LC3) pathway (Samuvel et al., 2022). Recent research determined that acute ethanol intake mediates mitochondrial depolarization and nuclear translocation of transcription factor EB (TFEB) to promote hepatocyte mitophagy (Samuvel et al., 2022). In ALD patients, hepatocytes can selectively eliminate ROS induced by alcohol through autophagy. Furthermore, autophagy can also remove oxidized lipid substances and damaged mitochondria, thereby reducing hepatocyte damage (Zhao et al., 2022; Khambu et al., 2017; Salete-Granado et al., 2023). Therefore, acute alcohol intake induces protective autophagy in hepatocytes to protect against oxidative stress and steatosis (Salete-Granado et al., 2023).

However, long-term alcohol intake gradually transforms alcohol-induced autophagy into inhibition. First, chronic alcohol intake can inhibit TFEB expression while increasing the activity of the mTOR, thereby inhibiting autophagy. Additionally, chronic alcohol intake can inhibit the Nrf2-ubiquinol cytochrome c reductase core protein 2 (UQCRC2) pathway and suppress mitophagy activity by downregulating adenosine monophosphate-activated protein kinase (AMPK) gene expression. Lu et al. demonstrated that mitophagy in hepatocytes is inhibited after chronic ethanol exposure, which may be closely related to the upregulation of DNA-dependent protein kinase catalytic subunits and activation of p53 (Lu et al., 2023a). Recent research determined that aryl hydrocarbon receptor (AhR) activation after alcohol intake in the liver, which is mediated by Protein Phosphatase 2 Regulatory Subunit-Bdelta (Ppp2r2d) to promote AMPKα dephosphorylation, leading to autophagy inhibition and mitochondrial dysfunction (Kim et al., 2022). Babuta et al. confirmed that alcohol intake upregulates miR-155 to reduce the protein expression of Lysosomal-associated membrane protein 1 and 2 (LAMP1 and LAMP2) in the liver, leading to impaired autophagy function of lysosomes and reduced autophagy flux (Babuta et al., 2019). Alcohol not only directly influences cellular autophagy, leading to liver cell damage, but also expedites the aging of liver cells by inhibiting autophagy. Lu et al. reported the significantly upregulated expression of zinc finger protein 281 (ZNF281) in alcohol-induced mice (Lu et al., 2023a). This protein inhibits hexokinase 2 (HK2) expression, resulting in a decline in mitochondrial autophagy-mediated through the HK2-PINK1/Parkin pathway, consequently promoting liver cell aging. Consequently, chronic alcohol intake is believed to impede the autophagy process in hepatocytes, exacerbating liver cell damage (Salete-Granado et al., 2023; Chao et al., 2018).

### 2.5 Cell death mode

**FIGURE 2:**
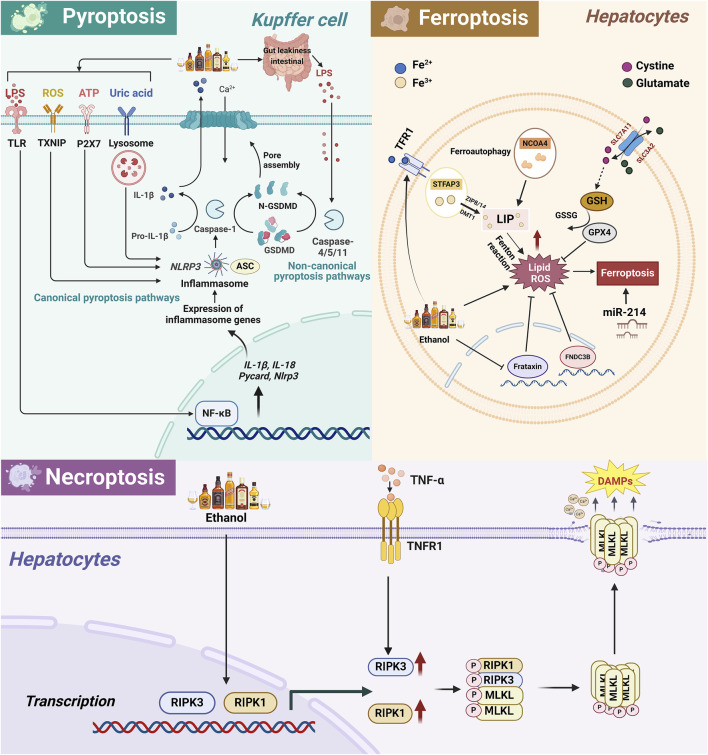
The related pathway mechanism of pyroptosis, necroptosis, and ferroptosis in the occurrence and development of ALD. 1) In ALD, alcohol metabolism leads to the activation of ROS/TXNIP, ATP/P2X7, uric acid/lysosome, LPS/TLR/NF-κB and other pathways, activating the classical pathway of KCs cell pyroptosis, namely, NLRP3, pro-caspase-1 and ASC assemble to form inflammasomes, activate caspase-1, and then cleave GSDMD, leading to cell membrane perforation and release of IL-18 and IL-1β, ultimately leading to cell pyroptosis. In addition, LPS intestinal leakage caused by alcohol intake can also directly activate the non-classical pathway mediated by Caspase4/5/11 in KCs, thereby cleaving GSDMD to induce cell pyroptosis and aggravate liver damage. 2) Alcohol intake enhances TFR1 expression and Fe3+ absorption in hepatocytes and also reduces Fe³⁺ to Fe^2^⁺ through STEAP3, transports it to the free iron pool via ZIP8/14 and DMT1. Simultaneously, NCOA4 promotes ferritin degradation, further increasing intracellular free Fe2+. Excessive Fe2+ triggers lipid peroxidation through the Fenton reaction, thereby triggering ferroptosis. Alcohol also causes an imbalance in the concentrations of glutamate and cystine inside and outside the cell through the SLC3A2/SLC7A11 channel, leading to GSH depletion and GPX4 inactivation, leading to hepatocyte ferroptosis. In addition, miR-214 exacerbates alcohol-induced hepatocyte damage by activating ferroptosis-related genes. 3) Necroptosis is a programmed cell death method that depends on the RIPK1/RIPK3 and MLKL axis. In ALD, the liver produces a large amount of proinflammatory cytokines such as TNF-α, which bind to TNFR1 to activate RIPK1/RIPK3 and then phosphorylate MLKL. Subsequently, p-MLKL forms oligomers in the cytoplasm and transfers to the cell membrane to create pores, destroying membrane integrity, causing cell rupture and death and releasing DAMPs, exacerbating liver inflammatory response and fibrosis. Abbreviation: ALD, Alcohol-Related Liver Disease; CYP2E1, Cytochrome P450 2E1; ROS, Reactive Oxygen Species; ATP, Adenosine triphosphate; LPS, Lipopolysaccharide; TLR, Toll-like receptors; TXNIP, Thioredoxin interacting protein; P2X7, Purinergic Receptor P2X, Ligand-Gated Ion Channel, 7; ASC, Apoptosis-Associated Speck-Like Protein Containing A CARD; NLRP3, NLR Family Pyrin Domain Containing 3; NF-κB, Nuclear Factor Kappa B; IL-1β, Interleukin 1 Beta; GSDMD, Gasdermin D; N-GSDMD, N-terminal fragment of GSDMD; TFR1, Transferrin Receptor; GSH, Glutathione; GPX4, Glutathione Peroxidase 4; GSSG, Glutathione disulfide; SLC7A11, Solute Carrier Family 7 Member 11; SLC3A2, Solute Carrier Family 3 Member 2; STFAP3, Six-Transmembrane Epithelial Antigen of Prostate 3; NCOA4, Nuclear Receptor Coactivator 4; ZIP8/14, Solute Carrier Family 39 Member 8/14; DMT1, Divalent metal transporter 1; LIP, Labile iron pool; FNDC3B, Fibronectin Type III Domain Containing 3B; RIPK1, Receptor Interacting Serine/Threonine Kinase 1; RIPK3, Receptor Interacting Serine/Threonine Kinase 3; MLKL, Mixed Lineage Kinase Domain Like Pseudokinase; DAMPs, Damage-associated molecular pattern; p-MLKL, Phosphorylated MLKL; TNF-α, Tumor necrosis factor; TNFR1, Tumor necrosis factor receptor 1.

The cell death mode in alcohol-related liver disease may involve various types, including pyroptosis, necroptosis, and ferroptosis. In recent years, these distinct modes of cell death increasingly recognized as being involved in the pathogenesis and progression of ALD and main mechanisms are summarized in Figure 2.

#### 2.5.1 Pyroptosis

Pyroptosis is a form of inflammatory cell death mediated by gasdermin, characterized by cell swelling and osmotic lysis, leading to cell membrane rupture and release of cellular contents, which play a role in a variety of pathological processes (Rao et al., 2022). In the canonical pathway of pyroptosis, intracellular pattern recognition receptors (PRRs) recognize PAMPs (Pathogen-Associated Molecular Patterns) or Damage-Associated Molecular Patterns (DAMPs), which can activate NLR family pyrin domain-containing 3 (NLRP3) and assemble with pro-caspase-1 and adaptor protein ASC to form inflammasomes. Subsequently, caspase-1 is activated, which in turn cleaves Gasdermin D (GSDMD), leading to cell membrane perforation and release of interleukin-18 (IL-18) and IL-1β, ultimately resulting in cell swelling and pyroptosis (Liu et al., 2024). In the atypical pathway, LPS activates caspase-4/5/11, which triggers cell membrane rupture and pyroptosis by cleaving GSDMD (Jin et al., 2023). A large amount of evidence shows that the activation of the pyroptosis system in ALD includes the classical pathway of caspase-1 activation induced by the synergistic action of ATP, uric acid and ROS, and the non-classical pathway of caspase-4/5/11 activation triggered by lipopolysaccharide (LPS) (Liu et al., 2022; Brahadeeswaran et al., 2023). PAMPs derived from the intestine and DAMPs from damaged hepatocytes can both induce pro-inflammatory cascades and participate in inflammasome-mediated pyroptosis in ALD (Szabo, 2017).

Current evidence has indicated that IL-1β and IL-18 levels, and caspase-1 expression are significantly elevated in the livers of AH patients and ALD mice (Miyata and Nagy, 2020). Previous research showed that alcohol inhibits Forkhead box protein O1, suppressing the expression of miR-148a, resulting in an overexpression of thioredoxin-interacting protein and activation of inflammasomes such as NLRP3. This leads to the pyroptotic destruction of liver cells and the release of NLRP3 inflammatory proteins, fostering liver fibrosis and liver damage (Gan et al., 2022; Heo et al., 2019). Thus, inhibiting pyroptosis through various pathways can mitigate the detrimental effects of alcohol on the liver. Kai et al. (2020) discovered that oroxylin A could boost peroxisome proliferator-activated receptor gamma coactivator 1 alpha/mitofusin 2 (PGC-1α/Mfn2) signaling, reduce ROS accumulation, prevent NLRP3 inflammasome activation, and block the classical caspase-1-dependent pyroptotic pathway, thereby reducing alcohol-induced liver cell damage. Another study reported that purines from hepatic macrophages could modulate the activation of NLRP3 inflammasomes through the purinergic receptor P2X7, exerting a significant influence on the progression of ALD and potentially serving as a fresh target for future ALD therapy (Le Dare et al., 2021). Ambade et al. (2019) demonstrated that inhibiting the C-C motif chemokine receptor 2/5 (CCR2/5) signaling pathway diminished alcohol-induced pyroptosis and reversed processes such as liver steatosis and fibrosis. Additionally, pyroptosis is closely linked to ERS. Research by Tien et al. (2023) demonstrated that pituitary tumor-transforming gene 1 (PTTG1) reduced ERS and hepatocyte pyroptosis, consequently alleviating liver injury. Tauroursodeoxycholic acid, an inhibitor of ERS, prevented pyroptosis in PTTG1-knockout LO2 cells, indicating a connection between ERS and pyroptosis.

#### 2.5.2 Ferroptosis

Ferroptosis is a recently recognized form of planned cell death dependent on iron. This process arises from iron ion-catalyzed and ROS-induced lipid peroxidation. Excessive alcohol intake induces oxidative stress, triggering the production of erythropoietin, which suppresses hepcidin generation. Simultaneously, it upregulates the expression of the transferrin receptor (TfR), intensifying iron absorption in liver cells and resulting in hepatic iron overload. This, consequently, exacerbates oxidative stress, leading to an excessive accumulation of ROS within hepatic cells. The central enzyme of the glutathione antioxidant system, glutathione peroxidase 4, becomes inactivated, leading to lipid peroxidation and eventual cell death (Li et al., 2022; Mueller et al., 2022). Given the crucial role of lipid peroxidation and the production of ROS in ferroptosis, identifying targets that can concurrently inhibit lipid metabolism disorders and iron-induced cell death has emerged as a pivotal focus in ALD research. Liu et al. observed that alcohol stimulation significantly inhibited the frataxin expression, leading to lipid peroxidation and ferroptosis in mice and in HepG2 cells. However, restoring frataxin expression decreased the susceptibility to ferroptosis, thereby mitigating ethanol-triggered liver damage (Liu et al., 2020). In another study, You et al. employed ALD modeling of mice with fibronectin type III domain-containing 3B (FNDC3B) gene knockout and discovered that the absence of FNDC3B significantly exacerbated lipid peroxidation (You et al., 2022). RNA sequencing analysis and the use of the iron-induced cell death inhibitor ferrostatin-1 demonstrated an association between FNDC3B and ferroptosis.

RAGE, a receptor for advanced glycation end products, plays a pivotal role in the development of ALD. Li et al. conducted a study and identified a positive correlation between RAGE levels and hepcidin, as well as TfR (Li et al., 2023). Long-term alcohol intake-induced hepatic oxidative stress and inflammatory responses can be alleviated, and liver iron metabolism can be improved by inhibiting RAGE expression. Recent research confirmed that miR-214 acts as a novel regulator of ferroptosis in ALD, enhancing chronic alcohol-triggered hepatocyte damage by transcriptionally activating ferroptosis-driving genes (Luo et al., 2023). In recent years, increasing evidence has suggested that traditional Chinese medicine and its active monomers can inhibit hepatocyte ferroptosis, exerting a protective effect against alcohol-induced liver injury. Dong et al. found that verbenalin alleviated alcohol-induced liver oxidative stress and mitochondrial dysfunction by modulating ferroptosis mediated by MDMX/PPARα (Dong et al., 2023). In another study, Song et al. demonstrated that ethanol or acetaldehyde-induced ferroptosis was closely associated with the autophagic degradation of nuclear receptor coactivator 4 -dependent ferritin. However, silibinin significantly attenuated ROS levels, ferritin degradation, and iron levels in hepatocytes, providing a novel therapeutic approach for alcohol-related liver injury (Song et al., 2022).

#### 2.5.3 Necroptosis

Necroptosis is a programmed cell death process dependent on the receptor-interacting serine/threonine-protein kinase 1/receptor-interacting serine/threonine-protein kinase 3/mixed lineage kinase domain-like pseudokinase (RIPK1/RIPK3/MLKL) axis. RIPK1 undergoes phosphorylation instead of ubiquitination, triggering the activation of RIPK3, which subsequently phosphorylates the mixed lineage kinase domain-like pseudokinase (MLKL). This leads to a structural alteration in MLKL, causing it to translocate to the plasma membrane, form pores, disrupt membrane integrity, facilitate an influx of extracellular water, and result in an efflux of intracellular K^+^. Ultimately, the cell swells and ruptures and the membrane potential are disturbed, culminating in necrotic cell death (Zhang et al., 2018c; Zhou et al., 2022a). Several studies demonstrated that RIPK3 expression was significantly increased in the livers of mice fed long-term ethanol and in liver biopsies of patients with ALD (Roychowdhury et al., 2013; Zhang et al., 2018a). Available evidence has revealed that the absence of RIPK3 inhibited necroptosis, lipid deposition, and mitochondrial ROS production following chronic alcohol intake (Zhou et al., 2022b). Notably, Previous studies have revealed that RIPK1 expression in mouse liver remains unchanged after ethanol feeding, and inhibition of RIP1 kinase by necrostatin-1 does not alleviate ethanol-induced hepatocyte injury (Roychowdhury et al., 2013). However, a recent study indicated that inhibiting RIPK1 can reduce alcohol-induced inflammatory gene expression and neutrophil recruitment (Wang et al., 2016). Zhou et al. reported that acute ethanol exposure increased the expression of RIPK1 and RIPK3, and increased the release of DAMPs to aggravate hepatocyte necroptosis (Zhou et al., 2022a). In addition, a recent study indicated that the active hepatoprotective ingredient tetramethylpyrazine inhibits the activation of RIPK1/RIPK3 necrosome and the phosphorylation of MLKL caused by alcohol intake, and exerts an anti-ALD effect by alleviating necroptosis (Zhou et al., 2022b). Interestingly, RIPA-56 and Necrostatin-1 (Nec-1, RIPK1 inhibitor) were confirmed to inhibit necroptosis and improve the inflammatory signaling pathway activated in mice fed high-fat diet (Majdi et al., 2020; Yang et al., 2019). Although the role of RIPK1 in ALD is currently controversial, more research is needed to further clarify.

MLKL not only holds a pivotal role in the terminal phase of necroptosis but is also intimately linked with the regulation of cellular functions. In a study by Wu et al. (2023a), bone marrow transplantation was performed between MLKL^−/−^ mice and wild-type mice, revealing that MLKL in the bone marrow could prevent alcohol-induced liver steatosis, inflammation, and other damage in ALD by affecting liver immune cell homeostasis and macrophage phagocytic capacity. Necroptosis mediated by MLKL is closely tied to liver fibrosis. Guo et al. (2022) demonstrated that MLKL deficiency diminished necroptosis in hepatocytes and curbed the stimulation of HSCs, consequently impeding the progression of liver fibrosis. Zhang et al. (2019) observed significant increases in the expression levels of RIPK3 and MLKL in hepatocytes with O-GlcNAc transferase (OGT) deficiency. Conversely, hepatocytes with acquired O-GlcNAc modification exhibited reduced necroptosis and liver fibrosis, indicating that OGT has the potential to inhibit necroptosis in hepatocytes and suppress the advancement of liver fibrosis. Hence, targeting the RIPK1-RIPK3-MLKL axis to inhibit necroptosis may emerge as a promising avenue for future research in the treatment of ALD.

### 2.6 Gut microecology

The intricate connection between the liver and the intestine constitutes the gut-liver axis. Changes in gut microbiota composition, microbial translocation, and disruptions in intestinal barrier integrity can influence CYP2E1 and lipid metabolism, and interfere with normal liver physiology, leading to pathological changes, such as liver inflammation and fibrosis (Maccioni et al., 2020). Thus, alcohol intake not only directly causes damage to liver cells but also compromises liver health by affecting gut microbiota and disturbing the gut-liver axis (Patel et al., 2022b; Jung et al., 2022). Hypoxia-inducible factor 1α (HIF-1α) is a nuclear protein closely associated with transcriptional regulation in inflammation. A study by Shao et al. reported that HIF-1α could modulate the expression of various factors associated with gut microbiota and intestinal barrier integrity (Shao et al., 2018). The absence of HIF-1α can result in intestinal wall damage, dysbiosis (e.g., increased *Akkermansia* abundance and decreased lactobacilli levels), heightened endotoxemia, and induced ALD. Besides mitigating alcohol-induced oxidative stress in the liver, studies have demonstrated that ALDH2 is pivotal in alcohol-induced intestinal permeability, intestinal epithelial barrier dysfunction, and gut bacterial translocation. *Porphyromonas gingivalis* (*P. gingivalis*) is a common pathogen contributing to periodontitis. As oral health problems worsen, the incidence of comorbid ALD with periodontitis is also increasing. Research has indicated that *P. gingivalis* can expedite alcohol-induced alterations in gut microbiota through the oral-gut-liver axis, thereby accelerating the progression of ALD (Gao et al., 2023). Therefore, preserving the stability of the gut-liver axis and its upstream pathways may serve as a crucial approach in future ALD treatment.

### 2.7 Liver immune response

The immune system of the liver assumes a pivotal role in mitigating the harm inflicted by ALD. Immune cells feature pattern recognition receptors (PRRs) on their surface, capable of binding to PAMPs and DAMPs triggered by ALD. This activation prompts the immune system to eliminate pathogens and deceased cells, consequently safeguarding the liver. However, owing to the intricate and diverse nature of PAMPs and DAMPs, which engage multiple signaling pathways, alcohol can disrupt the immune system through various avenues, potentially instigating immune cells to target the liver, thereby exacerbating liver damage (Dukic et al., 2023; Li et al., 2019; Kong et al., 2019). A large amount of evidence has shown that long-term drinking disrupts the intestinal barrier function, leading to impaired integrity of tight junction proteins and increased intestinal permeability. Subsequently, bacteria and LPS are translocated into the liver through the portal vein, activating the TLR4/NF-κB signaling pathway of liver Kupffer cells, and promoting the production of inflammatory cytokines such as TNF-α, IL-6 and IL-1β (Jiang and Schnabl, 2020).

Within the immune system, immune cells have perpetually played a pivotal role, and a growing body of research has focused on the potential mechanisms of immune cell-induced ALD damage. Neutrophils, the most numerous white blood cells in the human immune system, are intimately linked to AH due to their escalated numbers. In AH, there is a substantial surge in neutrophil activation. Influenced by diverse chemotactic factors such as C-X-C motif chemokine ligand 1 (CXCL1) and CXCL8, neutrophils adhere to hepatic sinusoidal endothelial cells by binding to vascular cell adhesion molecule 1 and hyaluronic acid, facilitating their migration, ultimately reaching the liver, and exacerbating the inflammatory response (Khan et al., 2023). This chemotactic migration is facilitated by lipocalin 2 (LCN2), and studies suggest that LCN2 may possess pro-inflammatory properties. Wieser et al. demonstrated that the expression of LCN2+ neutrophils in the liver of AH patients was increased compared with NAFLD patients (Wieser et al., 2016). In addition, compared with wild-type mice, ethanol-fed LCN knockout mice had significantly reduced serum transferase, and significantly improved inflammation and steatosis (Moschen et al., 2017).

**FIGURE 3:**
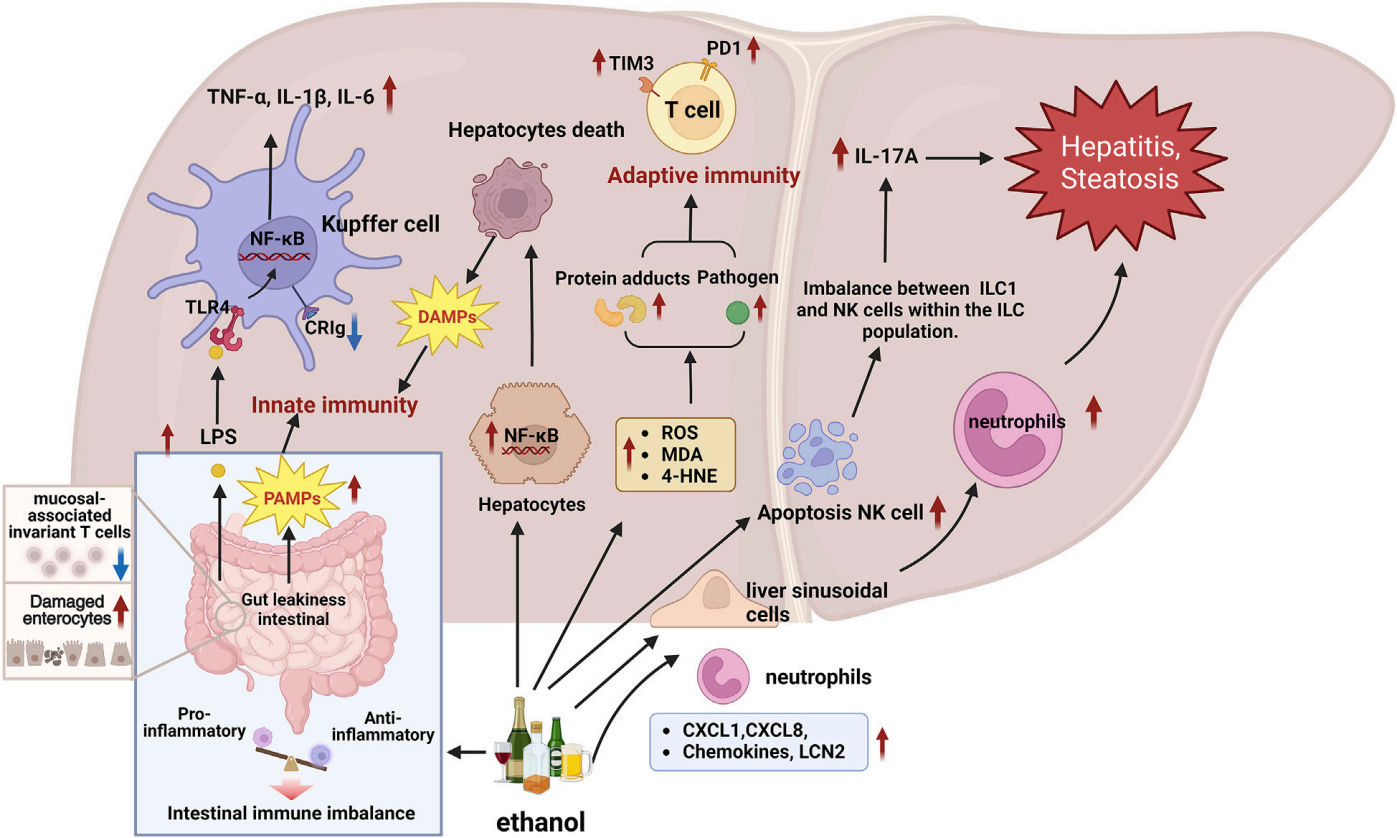
Molecular mechanisms of the immune response are involved in the progression of ALD. 1) Immune cells recognize PAMPs and DAMPs triggered by ALD through PRRs, initiating responses to clear pathogens and dead cells, which may also trigger immune cells to attack the liver, exacerbating liver damage. 2) Alcohol intake increases gut permeability and LPS leakage, LPS is translocated into the liver, activating the TLR4/NF-κB signaling pathway of liver Kupffer cells, and promoting the production of inflammatory cytokines such as TNF-α, IL-6 and IL-1β. 3) Chemokines CXCL1 and CXCL8 promote neutrophil adhesion to hepatic sinusoidal endothelial cells and migration to the liver, exacerbating liver inflammation. In addition, LCN2 promotes the chemotaxis of neutrophils. 4) In AAH patients, high expression of immune checkpoints PD1 and TIM3 on T cells may lead to impaired antibacterial immune response and decreased antibacterial activity of T cells and neutrophils. 5) Alcohol downregulates the expression level of CRIg on the surface of Kupffer cells, weakening the liver’s ability to phagocytize pathogens, thereby accelerating the progression of ALD. 6) Alcohol causes NK cell apoptosis, disrupts the balance between NK cells and ILC1 cells, increases the expression of IL-17A, and further aggravates liver inflammation and fibrosis. 7) Alcohol affects the intestinal immune balance, reduces mucosal-associated invariant T cells, increases intestinal susceptibility and causes intestinal damage, thereby destroying the intestinal-liver axis and aggravating ALD. Abbreviation: ALD, Alcohol-Related Liver Disease; CRIg, complement receptor of the immunoglobulin superfamily; CXCL1, C-X-C Motif Chemokine Ligand 1; CXCL8, C-X-C Motif Chemokine Ligand 8; DAMPs, Damage-associated molecular patterns; IL-17A, Interleukin 17A; ILC1, Group 1 innate lymphoid cells; LCN2, Lipocalin 2; NK, Natural killer; PAMPs, Pathogen-associated molecular patterns; PD1, Programmed Cell Death 1; PRRs, Pattern recognition receptors; TIM3, TIMP Metallopeptidase Inhibitor 3; KCs, Kupffer cells.

Apart from neutrophils, other immune cells in the liver also contribute to ALD damage. Research has indicated that antibacterial immune responses are impaired in patients with acute alcohol-associated hepatitis (AAH), which may be closely associated with the high expression levels of immune checkpoint receptors, programmed cell death protein 1 (PD1), and T-cell immunoglobulin mucin 3 (TIM3) on their T cells. However, blocking PD1 and TIM3 can restore innate and adaptive immunity in patients with AAH, enhancing the antimicrobial activity of T cells and neutrophils (Markwick et al., 2015). Evidence suggests that the complement receptor immunoglobulin (CRIg) superfamily member serves as a PRR involved in mediating phagocytosis. Duan et al. (2021) revealed that alcohol downregulated the expression level of CRIg on the surface of Kupffer cells. This reduction impairs the liver’s capacity to phagocytose pathogens, consequently expediting the progression of ALD. Chen et al.'s study demonstrated that excessive alcohol intake could induce apoptosis in natural killer (NK) cells, leading to an imbalance between type 1 innate lymphoid cells (ILC1) and NK cells within the ILC population (Cheng et al., 2023). This imbalance results in the heightened expression of IL-17A, subsequently driving processes such as alcohol-induced steatosis and inflammation, thereby expediting the onset of ALD. In addition to its direct effect on the immune system within the liver, alcohol can hasten the progression of ALD by affecting intestinal immunity and disrupting the gut-liver axis. Alcohol significantly reduces the number of mucosal-associated invariant T cells, heightens intestinal susceptibility, and induces intestinal damage, thereby advancing ALD (Riva et al., 2018). In general, Immune responses play a crucial role in the occurrence and development of ALD and the main mechanisms are shown in Figure 3.

### 2.8 Epigenetic modification

The term epigenetics pertains to alterations in gene function while retaining the same gene sequence. Common forms of epigenetic modifications encompass DNA modifications, noncoding RNA modifications, and histone modifications. An expanding body of research indicates that alcohol induces epigenetic modifications in diverse tissues and organs within the human body, ultimately leading to ALD. Among these, DNA methylation is one of the most common forms of DNA modification (Ajoolabady et al., 2022; Meroni et al., 2018). Studies revealed that chronic alcohol consumption inhibited the expression of tet methylcytosine dioxygenase (TET) proteins, resulting in the diminished production of 5-hydroxymethylcytosine, an emerging epigenetic modification. This leads to a decline and an increase in DNA methylation, consequently expediting liver cell apoptosis and causing harm to liver cells (Ji et al., 2019). Wang et al. (2019) conducted experiments and identified two methylation sites, namely, cg00510447 and cg26808293, in the TNF receptor superfamily member 12A (TNFRSF12A) gene with low methylation levels. These sites were predictive of a poor prognosis in patients with HCC who had a history of alcohol intake. The methylation status of this gene can be regulated by the methyltransferase DNA methyltransferase 3-like, exerting an influence on HCC development. Histone acetylation, another epigenetic modification induced by alcohol, also plays a significant role. Alcohol promotes the nuclear translocation of acetyl-CoA synthetase 2, heightening euchromatic histone lysine methyltransferase 2 acetylation, thereby accelerating fatty acid synthesis and driving hepatic lipid accumulation, ultimately leading to liver damage (Lu et al., 2023b).

MicroRNAs (miRNAs) are endogenous, small, noncoding RNA molecules, typically spanning 21–23 nucleotides, responsible for regulating gene expression at the translational level. Simultaneously, miRNAs represent the principal epigenetic regulatory factors involved in the pathogenesis of ALD. They interact with numerous pathways and feature intricate regulatory mechanisms. As such, there is a burgeoning body of research delving into the roles various miRNAs play in the onset of ALD. Previous studies found that chronic alcohol intake could significantly induce the expression of miR-155, miR-182, and miR-132 in hepatic tissues. However, microRNAs, such as miR-122 and miR-203, are significantly downregulated during the pathogenesis of ALD (Kong et al., 2019). Recently, Wang et al. uncovered that hsa-miR-1301-3p suppressed the expression of alcohol dehydrogenase 6 (class V) (ADH6), aldehyde dehydrogenase 5 family member A1 (ALDH5A1), and aldehyde dehydrogenase 8 family member A1 (ALDH8A1) within the alcohol oxidation pathway, effectively dampening alcohol-induced oxidative stress and mitigating liver damage (Wang et al., 2020). Additionally, miR-29c-3p, identified as a novel epigenetic regulatory factor, has been shown to enhance the activity of the ADH6 enhancer, consequently bolstering the expression of the ADH gene cluster, which includes alcohol dehydrogenase 1A (class I), alpha polypeptide (ADH1A), and alcohol dehydrogenase 1B (class I), beta polypeptide (ADH1B) (Chen et al., 2022). Luo et al. noted that alcohol downregulates hsa-miR-148a-3p through HNF4A, leading to reduced mRNA stability and the diminished expression of cytochrome P450 family 2 subfamily B member 6 (CYP2B6), thus contributing to the progression of ALD and causing liver damage (Luo et al., 2021).

### 2.9 Effects of other non-parenchymal cells

#### 2.9.1 Dysfunction of liver sinusoidal endothelial cells

Increasing evidence indicates that liver sinusoidal endothelial cells (LSECs) play a key role in the development of ALD. As the main non-parenchymal cells of the liver, LSECs have a unique fenestration structure and strong endocytic capacity, which are essential for maintaining liver homeostasis. LSECs have multiple functions such as anti-inflammatory, endocytic clearance, sinusoidal capillarization, and secretion of pro-angiogenic signaling factors. Therefore, impaired function is one of the key factors in the pathogenesis of ALD (Shetty et al., 2018; DeLeve, 2015). There is evidence that endotoxins and other related factors cause LSECs to stagnate in the G1/S phase and proliferate abnormally, thereby aggravating alcoholic liver damage (Nanji et al., 1994). It is reported that endothelial nitric oxide synthase (eNOS) in LSECs induces the production of nitric oxide (NO) to promote vasodilation, and support hepatocyte proliferation and liver regeneration (Nanji et al., 1996; Deleve et al., 2008). It is worth noting that ALD patients have LSEC differentiation and dysfunction, and impaired NO production will stimulate LSEC capillarization and dysfunction. In recent years, studies have found that sphingosine-1-phosphate (S1P) can activate eNOS, thereby increasing intracellular calcium concentration and NO content, maintaining LSECs activity and alleviating alcohol-induced liver damage (Zheng et al., 2006). Yang et al. (2021) showed that alcohol metabolism by CYP2E1 increases the acetylation of heat shock protein 90 (Hsp90) and reduces its interaction with eNOS, leading to reduced NO production and aggravated liver damage. However, overexpression of HDAC6 (Histone Deacetylase 6, HDAC6) can reverse the above process, repair LESCs function, and thus alleviate alcoholic liver damage. In addition, upregulated expression of TXNIP (Thioredoxin Interacting Protein, TXNIP) in LSEC has a protective effect in improving ALD. However, the loss of Txnip in LSEC leads to sinusoidal capillarization, downregulated NO production, and increased release of proinflammatory cytokines and adhesion molecules, and aggravates alcohol-induced liver damage, inflammation, and fibrosis (Jung et al., 2024).

#### 2.9.2 Activation of hepatic stellate cells

Many studies have shown that hepatic stellate cells (HSCs) are involved in the progression of ALD through various mechanisms. HSCs are located in the perisinusoidal space of the liver and are mainly responsible for storing vitamin A. They are also involved in multiple physiological functions of the liver, such as hepatocyte regeneration and liver immune regulation. However, under the influence of chronic alcohol, HSCs are activated by paracrine signals from apoptotic hepatocytes, LSECs, Kupffer cells (KCs), and infiltrating immune cells. HSCs express α-smooth actin (α-SMA) and increase the synthesis of type I and type III collagen, leading to extracellular matrix (ECM) deposition and promoting the progression of liver fibrosis (Khomich et al., 2019). Moreover, the activation of HSCs is closely related to oxidative stress and lipid peroxides caused by long-term alcohol intake (Friedman, 2008; Puche et al., 2013). Neuropilin-1 (NRP-1) is a growth factor co-receptor associated with HSC activation. Inhibition of this receptor has been shown to improve HSC recruitment and migration and block liver fibrosis (Cao et al., 2010). Recent evidence also shows that specific deletion of NRP-1 in HSC can alleviate alcohol-induced fatty liver hepatitis by regulating Igfbp3 and SerpinA12 signaling (Arab et al., 2020a). In addition, miRNA is a class of endogenous small non-coding RNA molecules. In recent years, it has been confirmed that it plays a key role in regulating metabolism, apoptosis and inflammation in ALD (Kumar et al., 2023). Wan et al. (2017) found that miR-34a content was significantly upregulated in ALD clinical patients and mice, and the mechanism may be to promote alcohol-induced liver fibrosis by reducing HSC aging. In addition to miRNA, long non-coding RNA XIST, as a competitive endogenous RNA of miR-29b, promotes HMGB1 expression, thereby enhancing ethanol-induced HSC autophagy and activation, thereby aggravating alcoholic liver fibrosis (Xie et al., 2019).

## 3 Therapeutic strategy for ALD

At present, alcohol abstinence remains the most fundamental treatment strategy for ALD at all stages. AH is the most severe acute form of ALD, and the mortality rate of patients with severe AH is as high as about 50%. Despite this, the drug treatment of AH is still mainly corticosteroids, which provide survival benefits for some patients with severe AH by reducing immune activation and blocking cytotoxic and inflammatory pathways (Ayares et al., 2022). In addition, Louvet et al. conducted a meta-analysis of data from 11 randomized controlled trials and found that corticosteroids can reduce the risk of death within 28 days of treatment (Louvet et al., 2018). Clinical and animal studies have confirmed that the tumor necrosis factor-α (TNF-α) inhibitor pentoxifylline treats AH and exerts anti-inflammatory effects by inhibiting phosphodiesterase.

(Singal and Shah, 2016). In addition, antioxidant therapy (such as N-acetylcysteine and Metadoxine) and nutritional support are also important components of the comprehensive treatment strategy for ALD (Singal et al., 2018). In recent years, emerging therapies for ALD have mainly focused on blocking inflammatory pathways, promoting liver regeneration, and restoring intestinal microecological balance (Sehrawat et al., 2020; Jophlin et al., 2024). These therapies provide new perspectives and hopes for the treatment of ALD. A summary of clinical trials for the treatment of alcoholic hepatitis is shown in Table 1.

**TABLE 1:** Current clinical trials for therapeutic agents for the treatment of ALD.

Pharmaceutical agent	Mechanism of action	Status	NCT number	References
Anakinra	IL-1 and other cytokine blockade	Phase 3, completed	NCT01809132	Szabo et al. (2022)
Canakinumab	IL-1β monoclonal antibody	Phase 2 Active Recruitment Status	NCT03775109	Vergis et al. (2024)
Obeticholic acid (INT-747)	FXR activation, bile acid agonist, anti-inflammatory	Phase 2, terminated	NCT02039219	Vergis et al. (2019)
Emricasan {IDN-6556}	Pan caspase inhibitor	Phase 2, completed	NCT02230670	Frenette et al. (2019)
DUR-928	Sulfated oxysterol, endogenous epigenetic regulator	Phase 2, completed	NCT03432260	Hassanein et al. (2024)
N-acetylcysteine	Antioxidant	Phase 3, completed	NCT00863785	Nguyen-Khac et al. (2011)
Metadoxine	Antioxidant	Phase 4, completed	NCT02161653	Higuera-de la Tijera et al. (2015)
F-652	Liver cell repair, anti-apoptotic, anti-inflammatory	Phase 2, completed	NCT02655510	Arab et al. (2020b)
G-CSF	Promote liver regeneration	Phase 4 Unknown status	NCT02971306	Singh et al. (2018)
*Lactobacillus rhamnosus GG*	Change in gut microbiome	Terminated	NCT01922895	Vatsalya et al. (2023)
*Probiotics*	Change in gut microbiome	Phase 4 Completed	NCT01501162	Han et al. (2015)
Fecal microbiota transplant	Gut–liver axis modification	Unknown status	NCT03091010	Pande et al. (2023)
HA35	Repair intestinal barrier function	Early Phase 1 Recruiting	NCT05018481	Wakil et al. (2023)
Bovine Colostrum	decrease the level of Endotoxemia and LPS	Phase 2, completed	NCT02265328	Yoon and Kim (2023)
Bovine colostrum (IMM-124E)	IgG against LPS and reduces bacterial translocation	Phase 2, completed	NCT01968382	Yoon and Kim (2023)
Rifaximin	Antibiotic	Phase 2, completed	NCT02116556	Jimenez et al. (2022)

### 3.1 Blocking inflammatory pathways

Liver inflammation and the resulting liver fibrosis/cirrhosis are the main pathological basis for the progression of liver disease. A variety of inflammatory mediators and cell adhesion molecules are involved in the regulation of inflammation, and the interactions between them together constitute a complex regulatory network (Gao et al., 2019a). Long-term alcohol consumption leads to liver inflammatory response, mainly immune cells recognize PAMP or DAMP through PRR, such as Toll-like receptor (TLR), and initiate transcriptional activation of proinflammatory mediators. Subsequently, it promotes the release of a variety of inflammatory cytokines, such as IL-1, TNF-α, IL-10, and neutrophil chemotactic factors (such as IL-8), recruit’s leukocytes in the liver, and amplifies the inflammatory cascade (Tang et al., 2012; Glaser et al., 2020). The above changes lead to mitochondrial damage and endoplasmic reticulum stress, interfere with liver metabolic responses, and aggravate liver damage (Dukic et al., 2023). Therefore, blocking the inflammatory pathway provides a new target for the treatment of AH.

Anakinra, as a recombinant IL-1 receptor antagonist, can block the biological activity of IL-1α and IL-1β, thereby inhibiting the inflammatory cascade (Szabo et al., 2022). However, in a recently completed multicenter, phase III randomized controlled trial (NCT04072822), the results have shown that compared with Anakinra combined with zinc treatment, the prednisone treatment group showed higher survival and lower incidence of acute kidney injury at 90 days in severe alcoholic hepatitis (SAH) (Gawrieh et al., 2024). The above results indicate that although Anakinra combination therapy may be beneficial in some cases, it does not show better efficacy than traditional corticosteroid treatment.

Current studies have demonstrated that oxidative stress and proinflammatory environment in hepatocytes activate apoptosis signal-regulating kinase 1 (ASK1), exacerbating liver inflammation, apoptosis and fibrosis (Polyzos et al., 2020). Selonsertib is an orally active small molecule inhibitor that can target and inhibit ASK1. In a phase 2 randomized open-label clinical trial, selonsertib was shown to reduce the extent of liver fibrosis in patients with NASH and stage 2–3 fibrosis (Loomba et al., 2018). However, a recent phase II clinical trial evaluating the efficacy of selonsertib combined with prednisolone in the treatment of SAH patients showed no improvement in liver function or mortality compared with prednisolone alone (Mathurin et al., 2018). Besides, there are many drugs targeting AH inflammation in clinical trials, including IL-1β–blocking Ab (Canakinumab), FXR agonist obeticholic acid (OCA), pan-caspase inhibitor (Emricasan), and endogenous epigenetic regulator DUR-928 (Peeraphatdit et al., 2018; Frenette et al., 2019; Hassanein et al., 2024).

### 3.2 Promote liver regeneration

There is increasing evidence that impaired liver regeneration is a key pathogenesis of AH and is closely related to disease progression and poor prognosis (Xiang et al., 2020b). *In vitro* and *in vivo* experiments have shown that long-term drinking may impair hepatocyte replication by affecting G1/S and G2/M transitions of the cell cycle (Clemens, 2007; Kuttippurathu et al., 2016). Clinical studies have confirmed that the number of Ki67^+^ and phosphorylated STAT3^+^ hepatocytes and cholangiocytes in patients with alcoholic cirrhosis is significantly decreased, indicating that hepatocyte proliferation is inhibited (Horiguchi et al., 2007). Other studies have shown that elevated liver progenitor cell markers are associated with hepatocyte proliferation inhibition and short-term mortality in AH patients (Dubuquoy et al., 2015; Sancho-Bru et al., 2012).

F-652 is a human IL-22 recombinant fusion protein composed of human interleukin 22 (IL-22) and human IgG2 Fc fragment, and its mechanism of action is similar to that of natural IL-22. IL-22 mainly activates the Jakl/Tyk2-STAT3 pathway by binding to the IL-22R1 and IL-10R2 heterodimer receptors, thereby exerting effects such as hepatocyte survival, liver regeneration, and reducing inflammation and oxidative stress (Xiang et al., 2020a). The results of a phase 2a open-label clinical trial study (NCT02655510) showed that F-652 was safe in the treatment of AH patients. In addition, the MELD score, serum aminotransferases and inflammatory cytokines of AH patients decreased significantly, while regeneration markers increased significantly (Arab et al., 2020b). Interestingly, previous studies have shown that liver injury histology and survival rate were improved after mobilizing bone marrow cells with granulocyte colony-stimulating factor (G-CSF) (Yannaki et al., 2005). Preclinical studies have found that G-CSF alleviates alcohol-induced liver damage and promotes hepatocyte proliferation in ALD mice (Cho et al., 2022). In a randomized controlled clinical trial (NCT02971306), the results have shown that G-CSF therapy can improve liver function and prolong survival time in SAH patients compared with standard treatment, and significantly reduce the MELD and MDF scores of SAH patients (Singh et al., 2018). However, there is currently some controversy about G-CSF therapy, and its efficacy and safety need to be verified through more clinical trials.

### 3.3 Regulates the gut-liver axis

The gut-liver axis is a two-way connection between the intestine and the liver. Long-term drinking can affect multiple links of the gut-liver axis, such as damaging the intestinal mucosal barrier function, inducing intestinal flora imbalance, and abnormal bile acid metabolism (Shim and Jeong, 2020; Albillos et al., 2020). A large amount of evidence has shown that long-term drinking damages the intestinal barrier function, leading to impaired integrity of tight junction proteins and increased intestinal permeability. Subsequently, bacteria and endotoxins are translocated into the liver through the portal vein, activating the TLR4/NF-κB signaling pathway of liver Kupffer cells, and promoting the liver inflammatory cascade and fibrosis development (Jiang and Schnabl, 2020). At the same time, long-term alcohol intake can also lead to intestinal flora imbalance, a decrease in beneficial bacteria, an increase in harmful bacteria, and activate PAMP-mediated immune and inflammatory responses through the gut-liver axis (Tilg et al., 2022; Raya Tonetti et al., 2024). In addition, more and more studies have confirmed that AH patients and animals are often accompanied by abnormal bile acid metabolism, which affects the integrity of the intestinal barrier and is closely related to the poor prognosis of the disease (Way et al., 2022). Therefore, regulating the function of the gut-liver axis provides a potential therapeutic strategy for the prevention and treatment of ALD.

Several clinical trials have shown that probiotics such as *Lacticaseibacillus rhamnosus LGG* improve acute or chronic alcoholic liver injury by regulating intestinal flora and repairing intestinal barrier function (Vatsalya et al., 2023; Han et al., 2015).

Rifaximin is a non-systemic antibiotic with broad-spectrum antibacterial activity and has high safety characteristics due to low intestinal absorption (Ponziani et al., 2017). Intestinal bacterial overgrowth is commonly found in patients with alcohol-related liver disease, whereas Rifaximin has been shown to improve the above processes in AH patients and exhibit therapeutic potential (Yang et al., 2024; Xie and Singal, 2023). A clinical study has demonstrated that rifaximin and steroids has a good safety profile for 90 days in patients with AAH compared with the steroid control group alone. Besides, the incidence of infection-related acute-on-chronic liver failure (ACLF) was lower in the rifaximin treatment group, and clinical complications were significantly reduced (Jimenez et al., 2022). Fecal microbiota transplantation (FMT) is an emerging treatment method that transplants intestinal microbiota from healthy donors into patients to rebuild the patient’s intestinal microecology and treat related diseases. In a phase 1 double-blind randomized clinical trial, Bajaj et al. have found that FMT therapy is safe. Furthermore, serum IL-6 and lipopolysaccharide-binding protein levels were significantly reduced and butyric acid/isobutyric acid was significantly increased in patients with AUD-related cirrhosis who received FMT compared with placebo. In addition, FMT can also reduce patients’ alcohol craving and alcohol consumption and produce favorable microbial changes (Bajaj et al., 2021). Moreover, animal experiments have also found similar phenomena. Mice treated with FMT have reduced ethanol acceptance, intake, and preference after bacterial colonization (Wolstenholme et al., 2022). In addition, FMT therapy can also significantly improve intestinal leakage, liver damage and inflammation caused by alcohol (Lamas-Paz et al., 2024). Philips et al. (2017) have demonstrated that FMT treatment significantly reduced the severity of SAH patients, reduced or resolved liver disease complications (such as ascites or hepatic encephalopathy), and improved 1-year survival compared with standard care. Besides, many drugs that regulate the gut-liver axis are in clinical trials, such as hyaluronic acid HA35, Bovine colostrum and IMM-124E that improve intestinal barrier function (Bellar et al., 2019; Sidhu et al., 2015).

## 4 Animal models

Three well-established animal models for ALD are presently available: the binge drinking model, the Lieber-DeCarli model, the NIAAA model, and the Tsukamoto-French model. In this review, we summarize these animal models.

### 4.1 Binge drinking model

The acute alcohol intragastric model can simulate the liver damage caused by binge ethanol in people with no drinking history. A single binge-drinking model developed by Carson and Stephen (4–6 g/kg, perfusion) can simulate blood alcohol levels, behavioral effects, and physiological changes in human binge drinkers and has been widely used (Carson and Pruett, 1996; Liu et al., 2021). Higher concentrations of ethanol and fasting before gavage resulted in gastrointestinal damage, more severe liver damage, and even death in mice (Shukla et al., 2013). Acute alcohol administration can significantly affect liver mitochondrial function, and induce oxidative stress and inflammatory responses. In addition, other studies have shown that liver damage caused by multiple EtOH binges is associated with changes in fat metabolism, hepatic oxidative stress and inflammation, intestinal damage, and endotoxemia (Ghosh Dastidar et al., 2018). A previous study found that mice were given 30% alcohol (6 g/kg) three times at intervals of 12 h. After the third alcohol gavage, obvious vesicular lipid droplet accumulation occurred in the mouse liver, accompanied by the generation of inflammatory lesions (Abdelmegeed et al., 2013).

Due to the short modeling time and simple operation, the acute alcoholic liver injury model has great application space in studying the pathogenesis of acute alcoholic liver injury and developing related nutraceutical and therapeutic agents (Lai et al., 2024). However, this model has certain limitations: it only stays in the early stage of ALD and only causes mild increases in serum

Alanine aminotransferase (ALT), aspartate aminotransferase (AST) and mild steatosis, which limits its wide application (Ghosh Dastidar et al., 2018; Bertola et al., 2013a). Tien et al. (2023) demonstrated that the levels of Pituitary tumour transforming gene 1 (PTTG1) in the serum of patients with acute alcoholic liver injury were significantly attenuated. Further results of a single binge drinking mouse model (5 g/kg, 50%) showed that compared with wild-type mice, PTTG1 KO mice had more severe acute alcoholic liver injury and were accompanied by hepatic ERS aggravation and hepatocyte pyroptosis. A recent study indicated that mitochondrial ALDH2 knockout mice are more susceptible to binge drinking-mediated liver damage due to increased oxidative stress, intestinal leakage, and endotoxemia than WT mice (Rungratanawanich et al., 2023).

### 4.2 Lieber-DeCarli model

One of the earliest and most frequently employed experimental models for investigating ALD in rodents is the Lieber-DeCarli model. Adult C57BL/6 mice, aged 8–11 weeks, are selected for this model. They are administered a control liquid diet comprising 15% protein, 36% fat, and 49% carbohydrates, in addition to essential vitamins and minerals. The carbohydrates, constituting 36% of the total caloric content, are substituted with alcohol to formulate the Lieber-DeCarli diet. The modeling process of Lieber-DeCarli entails a 1-week adaptation phase, with alcohol concentrations gradually escalating from 0% to 5% w/v. After this adaptation period, the mice are maintained on the Lieber-DeCarli diet for a span of 4–12 weeks. The outcomes of this model indicate that mice fed for 4 weeks post-adaptation exhibit mild hepatic steatosis (fatty liver) along with a modest inflammatory response. At the 12th week, the mice demonstrate significant hepatic steatosis, with blood alcohol concentrations (BACs) reaching 100–150 mg/dL, accompanied by mild liver injury, albeit without apparent liver fibrosis (Zhu et al., 2022).

The strengths of the Lieber-DeCarli model lie in its cost-effectiveness, straightforward equipment requirements, and ease of operation, leading to reduced mouse mortality rates and substantial cost savings. Nevertheless, there are key considerations concerning this modeling approach. First, the feed is in liquid form, necessitating the utilization of a specialized liquid diet feeding tube for administration and daily cleaning. Additionally, due to the high volatility of alcohol in liquid form, preparing fresh feed each day is recommended. Because mice are nocturnal, it is advisable to change the feed at around 4 p.m., rendering this modeling approach somewhat time-intensive and laborious. Furthermore, as this model does not require additional water supply, transitioning from a liquid diet to solid food with water may affect the mice’s physiological state. This modeling approach rarely induces significant liver inflammation or fibrosis, which limits the study of advanced stages of ALD (Zhu et al., 2022; Guo et al., 2018).

Kumar et al. utilized the Lieber-DeCarli model to observe the upregulation of miR-96 and Shh in ethanol-fed mice. Simultaneously, the study also revealed a significant upregulation of miR-96, TGF-β1, SHH, and GLI2 in AH patients. These findings suggest a close association between the gene expression patterns in ethanol-fed mice and AH patients (Kumar et al., 2023). A recent study indicated a pronounced downregulation of PRMT6 expression in liver samples from patients with ALD and mice on the Lieber-DeCarli diet, which correlated inversely with disease severity. Further evidence demonstrated that alcohol-induced Prmt6 deficiency promoted alcohol-related fibrogenesis by reducing integrin methylation and increasing pro-fibrotic signaling in macrophages (Schonfeld et al., 2023).

### 4.3 NIAAA model

The chronic alcohol feeding (10 days) plus single binge (NIAAA) model, established by Gao’s team in 2010, entails administering the Lieber-DeCarli ethanol diet to male mice aged 8–10 weeks and female mice aged 10–12 weeks for a period of 10 days. On the 11th day, the mice receive an intragastric administration of ethanol solution (5 g/kg body weight). The results of this model reveal high BACs in mice, along with significant elevations in serum ALT and AST levels, both peaking at 9 h after administration. An increased infiltration of neutrophils and hepatic steatosis is also observed. However, the NIAAA model has certain limitations as it is unable to replicate the severe inflammatory and fibrotic pathological features of AH patients. Furthermore, the modeling process requires specialized feeding equipment, with experimental animals being individually housed or paired in a cage, resulting in increased experimental costs.

Recently, Ma et al. (2023) found a significant decrease in dynamin-related protein 1 (DRP1) levels and an increase in hepatic giant mitochondria accumulation in liver samples from ALD patients and NIAAA model mice. Further mechanistic investigations revealed that hepatic DRP1 deficiency led to increased mtDNA levels and mitochondrial dysfunction, resulting in the activation of the cGAS-STING-interferon signaling pathway and exacerbating alcohol-induced liver injury. Another study showed the significant activation of ABL kinase in liver samples of ALD patients, as well as in the liver tissues of NIAAA model mice. Experimental evidence further suggests the alcohol-induced activation of ABL2 promotes ALD by upregulating HIF-1α and subsequent PPARγ expression, suggesting that inhibiting ABL2 could be a promising therapeutic target for ALD treatment (Malnassy et al., 2023).

### 4.4 Chronic combined with binge model

Some epidemiological studies have shown that drinking patterns also significantly affect the pathogenesis of human ALD (Hatton et al., 2009). There is evidence that mixed drinking patterns are significantly associated with an increased risk of ALD (Stranges et al., 2004). The chronic superimposed binge drinking model is closer to the human drinking pattern and better simulates the development of alcoholic liver disease. Bertola et al. (2013b) have confirmed that compared with simple chronic or binge drinking, chronic plus binge drinking ethanol feeding synergistically upregulates the expression of IL-1β and TNF-α in the liver and induces the accumulation of neutrophils in the liver and elevated serum transaminases. A large amount of evidence shows that intrahepatic inflammatory cell infiltration is a significant clinical feature of hepatitis and is closely related to the severity and mortality of ASH patients (Feng et al., 2024; Khan et al., 2023).

Xu et al. (2015) have demonstrated that chronic alcohol feeding for 8 weeks + 1 Binge or chronic alcohol feeding for 8 weeks + n Binge models induce more severe ASH than short-term 10 days + 1 binge (NIAAA model). Moreover, this model mimics some aspects of early steatohepatitis in patients with AH, including elevated serum ALT and AST levels, AST to ALT ratio >2, severe steatosis, neutrophil infiltration, and mild fibrosis. Another study found that in the chronic plus binge ethanol feeding model, TLR2 and TLR9-mediated MyD88-dependent pathway activation led to the production of neutrophil recruitment chemokine CXCL1, exacerbating liver inflammatory infiltration (Roh et al., 2015). Overall, Chronic Combined with Binge Mode can simulate the drinking pattern of most patients with liver disease and reproduce some histological and molecular characteristics of clinical ASH patients.

### 4.5 Tsukamoto-French model

The transoral feeding Tsukamoto-French (TF) model necessitates the implantation of an esophageal catheter in rats, allowing for the direct infusion of alcohol-containing liquid feed (with alcohol accounting for 47% of the caloric intake) using a liquid pump. Unlike other administration methods, the TF model overcomes the innate aversion to alcohol often observed in rodents, ensuring better control over the alcohol intake of each experimental animal and reducing experimental errors. It is important to note that the TF model enables straightforward adjustment of the nutritional composition of the feed to create specific liver disease models. For example, combining alcohol feeding with a high-fat diet (with fat accounting for 25% of the caloric intake) in rats for 30 days can lead to significant liver fibrosis (Lamas-Paz et al., 2018). Furthermore, even with basic TF feeding, the BACs in experimental mice can be 2 to 3 times higher than those achieved by the oral administration of ethanol in drinking water, highlighting that the TF model can induce more pronounced alcohol intoxication. A substantial body of research has demonstrated that the imbalance of intestinal flora and its metabolites plays a crucial role in the pathogenesis of ALD. In a previous study, Chen et al. found that gut microbiota dysbiosis was common in ALD patients and the mouse model (Tsukamoto-French), resulting in a decreased capacity to synthesize saturated long-chain fatty acids (LCFA) from the microbial composition and a reduced proportion of *Lactobacillus* species (Chen et al., 2015).

However, the TF model has several limitations. First, the insertion of the gastric esophageal catheter demands the experimenter to possess strong technical skills and extensive experience. Additionally, postoperative care is critical, necessitating adequate training for the experimental staff. Furthermore, the gastric catheter and other experimental equipment require meticulous disinfection, sterilization, and proper storage. For instance, infusion sets are typically stored *in situ* for 2–4 months after use to minimize equipment contamination, which may affect experimental outcomes or result in animal mortality. Consequently, the establishment of the TF model entails high costs and personnel expenses, which not all laboratories can accommodate.

**FIGURE 4:**
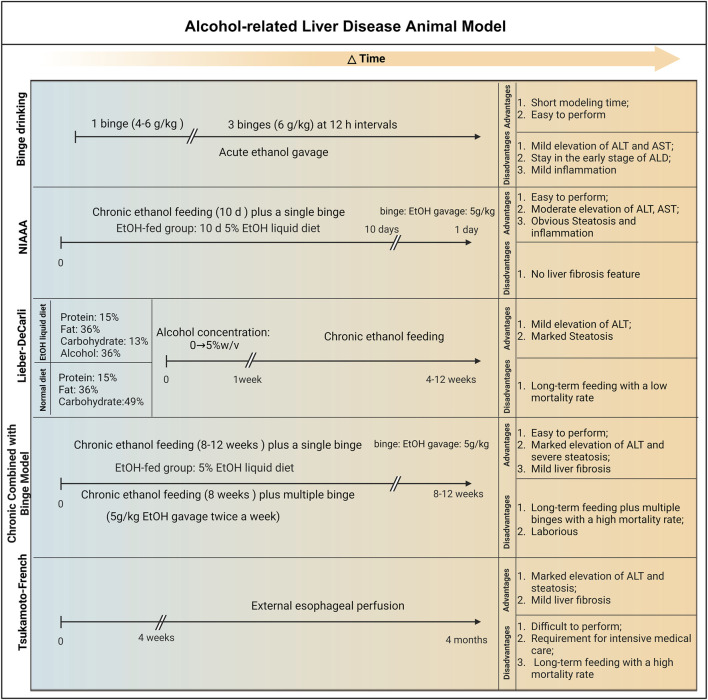
The establishment and advantages and disadvantages of animal models of ALD. 1) Binge Drinking Model: Animals are given 4–6 g/kg alcohol by gavage once or 6 g/kg alcohol by gavage three times within 12 h. This model is simple to operate and can cause mild elevation of serum transaminases and liver inflammation, reflecting only some liver lesions in the early stages of alcohol abuse. 2) NIAAA Model: Chronic alcohol feeding for 10 days (5% alcohol liquid feed) + 1 binge (4–6 g/kg alcohol gavage). This model combines chronic alcohol intake with a single binge, and can simulate chronic-acute alcoholic liver injury and steatosis in a relatively short period of time and moderately increase liver enzyme levels such as ALT and AST. In addition, this model does not cause a high mortality rate in the short term; however, this model cannot replicate the pathological characteristics of fibrosis. 3) Lieber-DeCarli Model: Feeding liquid alcohol feed with alcohol concentration gradually increasing from 0% to 5% w/v during a 1-week adaptation period, followed by 4–12 weeks of 5% w/v liquid alcohol feed. This model can induce mild elevation of liver enzymes such as ALT and obvious fatty degeneration, and is suitable for long-term chronic alcoholic liver disease research; however, the modeling method rarely causes severe liver inflammation or fibrosis. 4) Chronic Combined with Binge Model: 5% alcohol liquid feed feeding for 8–12 weeks + 1 binge (5 g/kg alcohol gavage) or 5% alcohol liquid feed feeding for 8 weeks + multiple binge (5 g/kg alcohol gavage). This model can better simulate the progression of human alcoholic liver disease, manifested by significant elevation of liver enzymes such as ALT, severe fatty degeneration, hepatitis and moderate liver fibrosis; however, this model increases the workload and leads to a high animal mortality rate. 5) Tsukamoto-French Model: An esophageal catheter is implanted in the body, and alcohol-containing liquid feed is directly introduced through a liquid pump (4 weeks–4 months). This model can induce significant elevation of liver enzymes such as ALT, severe fatty degeneration and moderate liver fibrosis; however, this model requires high technical difficulty, high animal breeding conditions and high cost, which limits its promotion and application. Abbreviation: NIAAA, National Institute on Alcohol Abuse and Alcoholism; EtOH, ethanol; ALT, Alanine Transaminase; AST, Aspartate Transaminase.

In summary, animal models of ALD are classified according to disease progression stages as shown in Figure 4. The acute alcohol binge drinking model is easy to operate and can induce mild serum aminotransferase elevations and mild inflammation. The levels of ALT and AST in NIAAA model mice were significantly increased, as well as marked hepatic steatosis and inflammatory response. The Lieber-DeCarli model can induce obvious liver steatosis, mild elevated serum transaminases and a small amount of liver inflammation. The Chronic Combined with Binge model can induce more severe liver steatosis, hepatocellular damage and infiltration of liver neutrophils. Chronic alcohol feeding combined with multiple high-dose alcohol gavages can simulate the drinking patterns of most patients with liver disease and are highly similar to the pathogenesis of human ALD. The Tsukamoto-French model can cause more severe fatty liver, liver inflammation and damage, but the complex operation and strict requirements for animal rooms limit its promotion. These models can be used to explore the pathogenesis of ALD at different stages or different degrees of severity, and provide research tools for discovering new drug targets with potential for the treatment of ALD.

## 5 Conclusion

The pathogenesis of ALD is intricate. However, the medical community recognizes a discernible causal relationship between oxidative stress, lipid deposition, ERS, cellular autophagy, the liver immune response, organ crosstalk (including the gut-liver axis), epigenetic regulation primarily by noncoding RNA, and various cell death pathways, encompassing pyroptosis, ferroptosis, and necroptosis, in the progression of ALD. Currently, abstinence, corticosteroids, and nutritional therapy remain conventional therapeutic interventions for ALD. However, some emerging therapies for ALD have shown promising outcomes, including the inhibition of inflammatory pathways (e.g., anakinra), the promotion of liver regeneration (e.g., IL22 agonist, F-652), and restoration of the normal microbiota (e.g., probiotics and rifaximin). With increasing interest in ALD drug development from pharmaceutical companies and funding agencies, we may anticipate the emergence of more novel therapies for treating ALD in the future. With the escalating research focus on ALD, conventional modeling approaches for ALD have seen gradual refinement and expansion. These novel models are anticipated to provide further insight into the pathogenesis of ALD, illuminate the reciprocal interplay between the liver and other organs, and contribute to advancements in the prevention and treatment of ALD.
